# A Case of Palmoplantar Keratoderma in the Constellation of Connective Tissue Diseases

**DOI:** 10.7759/cureus.56531

**Published:** 2024-03-20

**Authors:** Ishan Verma, Amol H Dube, Sunita Kumbhalkar, Keshao Nagpure, Gitesh Sawatkar, Sachin R Chuadhari, Ashwini Umredkar

**Affiliations:** 1 General Medicine, All India Institute of Medical Sciences, Nagpur, Nagpur, IND; 2 Dermatology, All India Institute of Medical Sciences, Nagpur, Nagpur, IND; 3 Pathology, All India Institute of Medical Sciences, Nagpur, Nagpur, IND; 4 Radiodiagnosis, All India Institute of Medical Sciences, Nagpur, Nagpur, IND

**Keywords:** anti-golgi apparatus ab, topical tacrolimus 0.1%, normal cpk, anti-pm/scl ab, hypomyopathic dermatomyositis, overlap syndromes, low-dose methotrexate, palmoplantar keratoderma

## Abstract

Overlap syndrome is a clinical challenge and brings together a wide range of treatment options for the treating physician. Addressing each and every complaint of the patient is crucial.

A 50-year-old female patient presented with skin thickening, blackening, and hyperkeratosis; dysphagia; joint pain; features of myopathy; Raynaud’s phenomenon; and dry mouth. Inflammatory markers were raised along with a positive antinuclear antibody (ANA) with Golgi apparatus pattern, anti-Sjögren’s-syndrome-related antigen A (anti-SSA)/Ro60 3+, anti-SSA/Ro52 3+, and anti-PM/Scl 2+ antibodies that suggested overlap syndrome. Although the patient had no respiratory complaints, a unique interstitial lung disease (ILD) pattern was noted during the evaluation. Skin manifestations were puzzling, but the histopathology analyses of skin biopsies taken from two different sites revealed distinguishing features of cutaneous lupus and dermatomyositis. Treatment with hydroxychloroquine, pilocarpine, nifedipine, methotrexate, and topical tacrolimus produced a dramatic improvement in the clinical features.

This case highlights subtle and florid features of different autoimmune diseases. The hyperkeratotic skin changes were the most striking feature, but the whole evaluation process unveiled many rare presentations of known autoimmune conditions that can open doors to new areas of our understanding toward connective tissue diseases (CTDs). Our case report demonstrates significant heterogeneity in the ANA patterns, ILD patterns, clinical manifestations, and treatment approaches.

## Introduction

The world of rheumatology is full of intricacies. Autoimmune diseases are classic examples of the famous saying, “Exceptions are always there!” We present a case of palmoplantar keratoderma with multiple somatic complaints and some elements from many connective tissue diseases (CTDs) but which could be classified in none. There were some unexpected clinical and biochemical features. Simultaneous skin features of dermatomyositis and cutaneous lupus, unclassifiable interstitial lung disease (U-ILD), and unusual antinuclear antibody (ANA) patterns are key highlights of this case report. Furthermore, the treatment strategy resulted in a positive outcome. Through this case, we would like to highlight the importance of clinical examination and how a broader vision can address the patient’s detailed complaints.

## Case presentation

**Figure 1:**
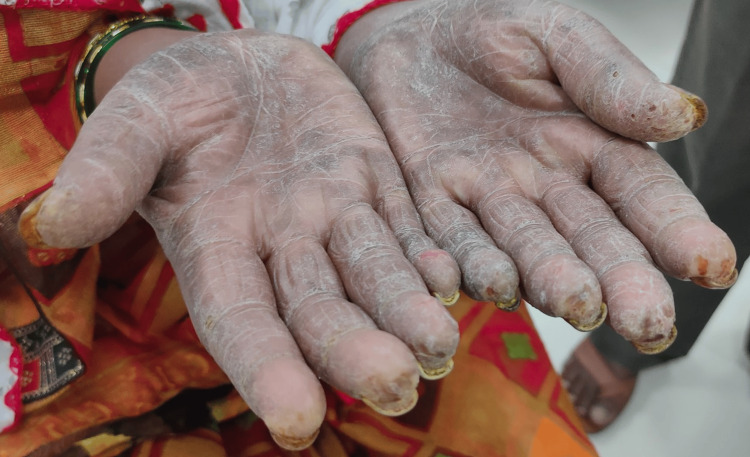
Digital ulcers, fissured fingers, and erythematous scaly plaques over the palms.

**Figure 2:**
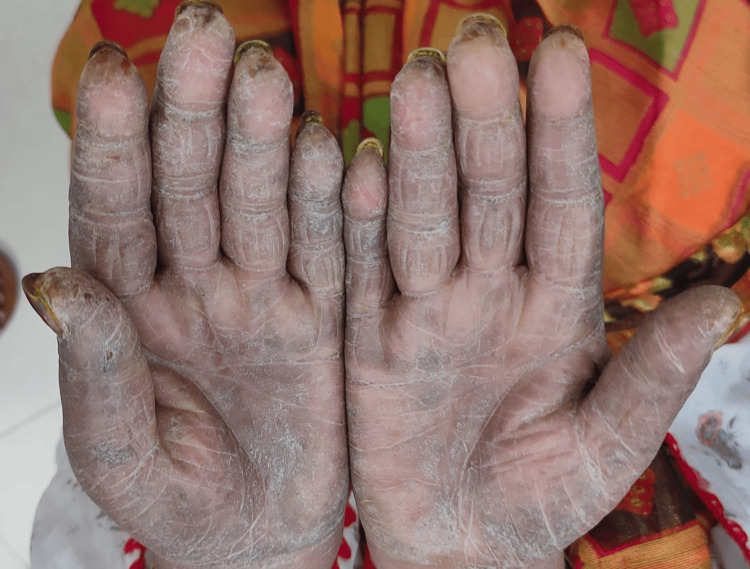
Digital ulcers, fissured fingers, and erythematous scaly plaques over the palms.

**Figure 3:**
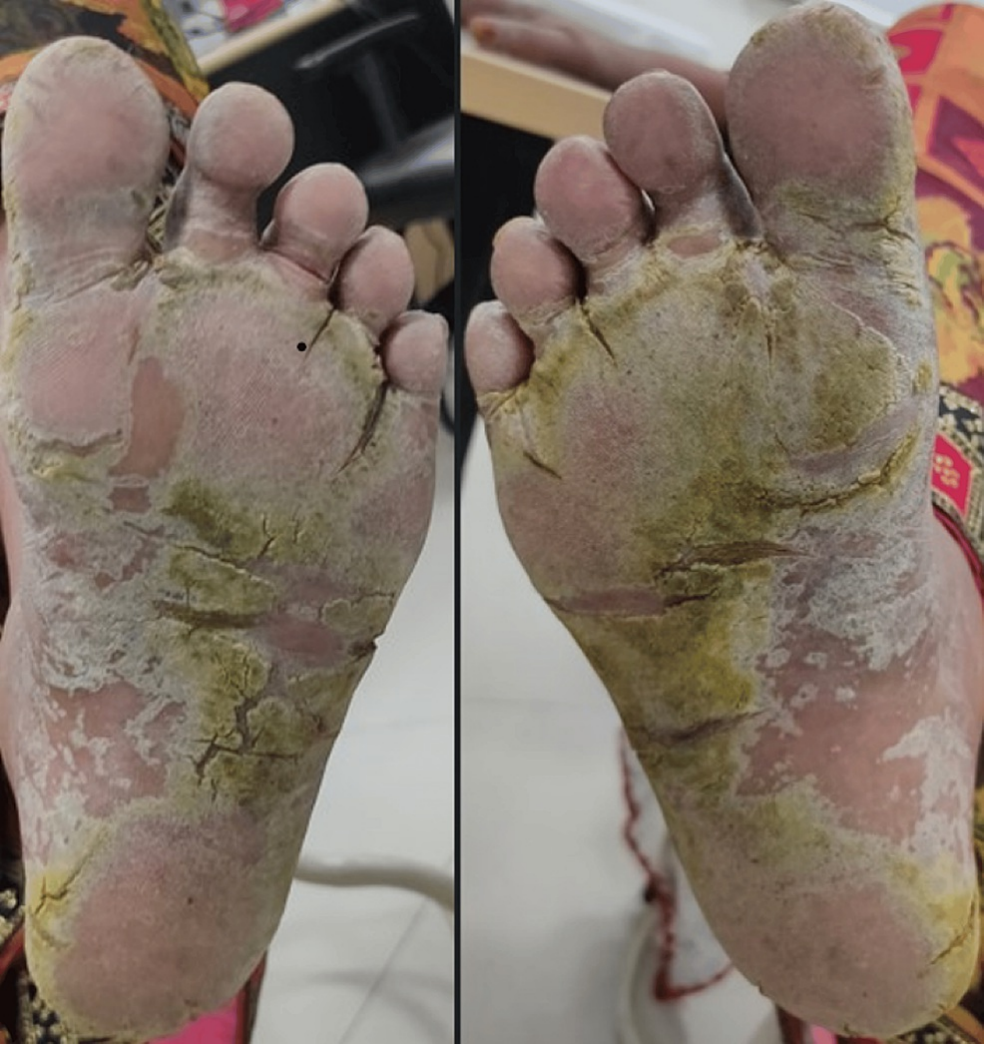
Scaly plaques and fissures over both soles.

**Figure 4:**
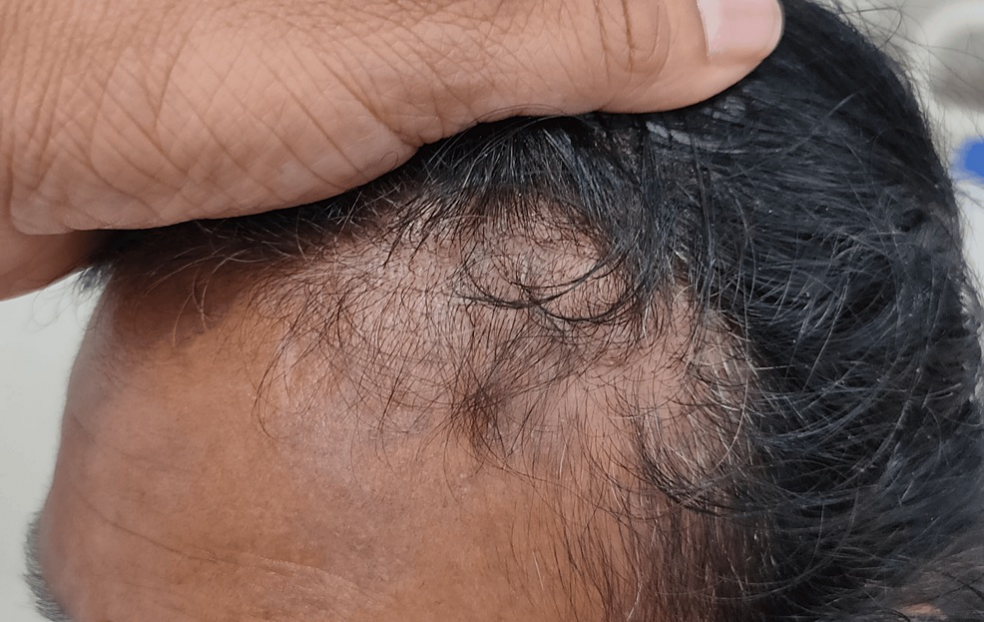
Alopecia.

**Figure 5:**
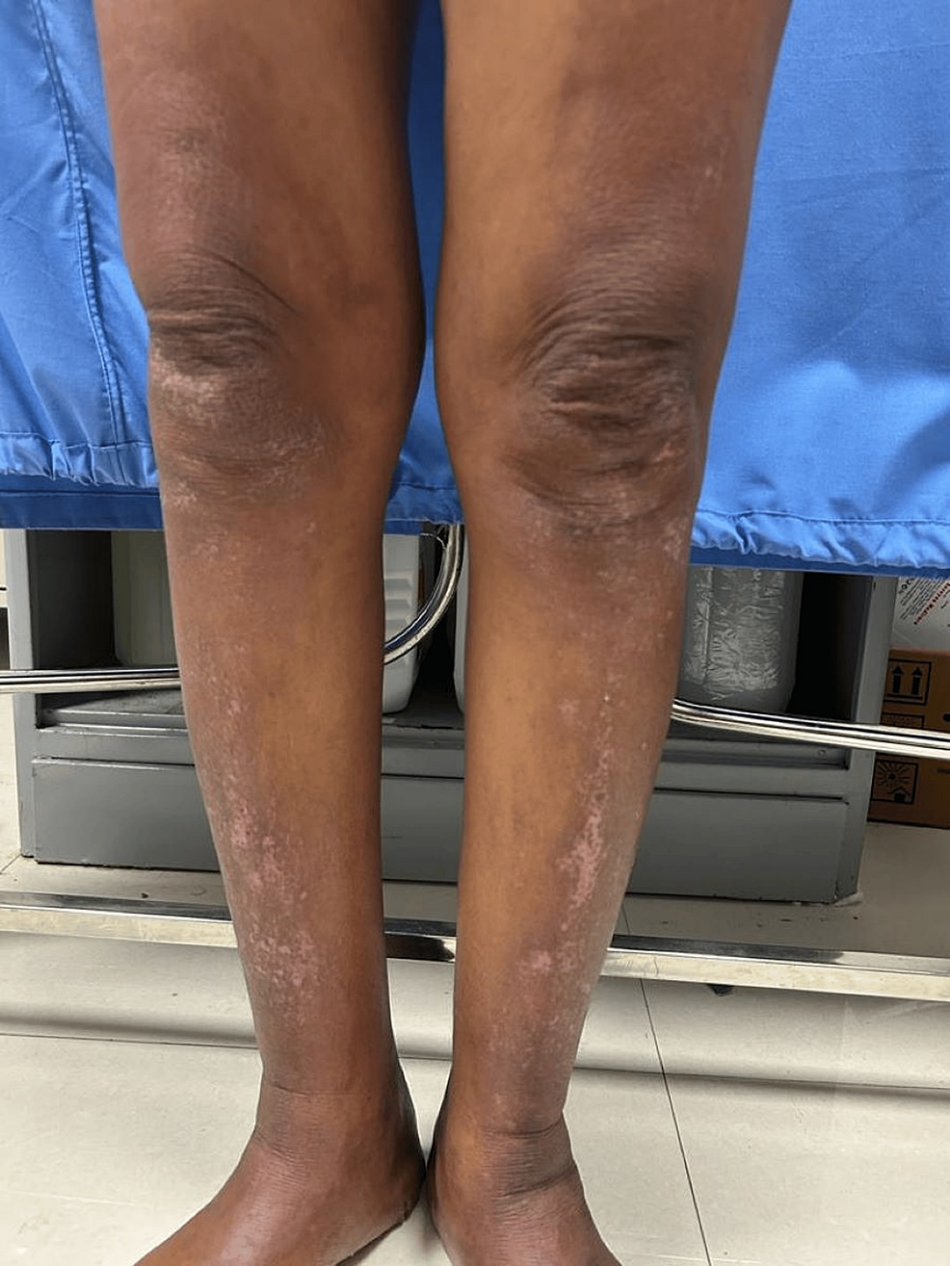
Salt-and-pepper pigmentation of both legs.

A 50-year-old female without comorbidities arrived in the medicine department with complaints of blackish discoloration of both hands (Figures 1, 2) and the feet; erythematous scaly plaques over both palms (Figures 1, 2) and soles (Figure 3); multiple, discrete, ill-defined scaly erythematous and psoriasiform plaques on both hands, the elbows, the feet, the upper legs extending up to buttocks, the upper part of the chest and upper back; plaques in her nail beds (Figures 1, 2), fissured fingers (Figures 1, 2), increased hair loss (Figure 4), facial puffiness, pain and swelling of the small joints of both hands and the feet with morning stiffness; mild skin thickening over the forearms, hands, and feet; difficulty in deglutition for both solids and liquids;’ difficulty in combing hair; difficulty in getting up from a squatting position; photosensitive skin rashes over the face; whitish/bluish discoloration of the finger tips on exposure to cold; digital tip ulcers (Figures 1, 2); low backache; and salt-and-pepper pigmentation over both legs (Figure 5) lasting for one year. She reported a history of episodes of recurrent oral ulcers, red eyes, and loose stools. The above clinical data are sufficient to suspect that connective tissue disease is present.

On examination, her pulse was 86 beats/min, blood pressure (BP) was 130/80 mmHg, and arterial oxygen saturation was normal. Systemic examination was normal. Based on the above symptomatology, the following differential diagnoses were suspected: (a) overlap syndrome with features of systemic sclerosis, systemic lupus erythematosus (SLE), and inflammatory myopathy; (b) SLE; (c) antisynthetase syndrome (based on her mechanic’s hands, arthritis, and Raynaud’s phenomenon); (d) reactive arthritis (based on her history of loose stools and keratoderma blennorrhagica); and (e) psoriatic arthritis (based on low backache, arthritis, and skin features).

Investigation table (Table 1) shows the following: X-ray of the pelvis did not reveal any sacroiliitis. MRI pelvis was normal. Fundus examination was normal. ANA immunofluorescence assay (IFA) showed 1:1000 3+ nucleolar and a Golgi apparatus pattern. This is a unique pattern that is rarely found. ANA immunoblot was done that showed anti-Sjögren’s-syndrome-related antigen A (anti-SSA)/Ro60 3+, anti-SSA/Ro52 3+, and anti-PM/Scl 2+ consistent with overlap syndrome. X-ray of her hands revealed nonerosive arthritis.

**Table 1:** Investigation table. RA factor: rheumatoid factor; ANA: antinuclear antibody; ELISA: enzyme-linked immunosorbent assay; LDH: lactate dehydrogenase; CRP: C-reactive protein; IFA: immunofluorescence assay; CPK: creatinine phosphokinase; FEV1/FVC ratio: forced expiratory volume in the first one second to the forced vital capacity of the lungs; TSH: thyroid-stimulating hormone;

Investigations	Patient’s value	Reference range
Hemoglobin	10.3 g/dL	12–15 g/dL
Urine routine microscopy	Protein 1+	Nil
RA factor	101.4 IU/ml	<14 IU/ml
ANA ELISA	>12	<1 (negative), <1.2 (positive)
LDH	258 U/l	135–214 U/l
CRP	6.17 mg/L	<5 mg/L
TSH	8.09 mIU/mL	0.27–4.20 mIU/mL
ANA IFA	1:1000	<1:80
Total CPK	45 U/L	26–192 U/L
Schirmer’s test	9 mm and 5 mm	>10 mm in 5 minutes
Pulmonary function test – FEV1/FVC ratio	FEV1/FVC 94.6% (pre bronchodilator) and 88.2% (post bronchodilator) shows restrictive pattern	60.2%–90.3%

**Figure 6:**
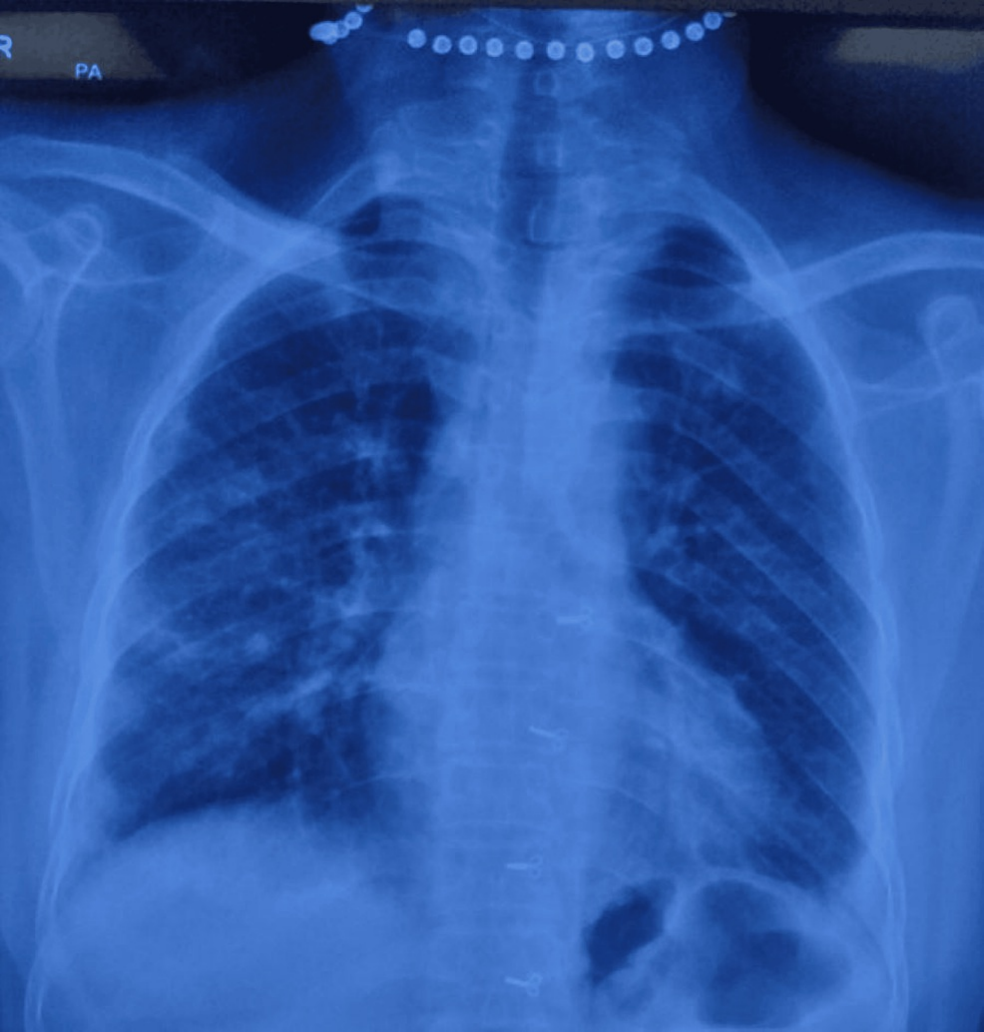
Chest X-Ray showing reticular opacities and left-sided pleural effusion.

Hydroxychloroquine tablet and sulfasalazine tablet were started, after which joint pain improved, but the proximal myopathy and keratoderma did not. Prednisolone tablet was started at 1 mg/kg/day, after which her proximal muscle weakness significantly improved. Although there were no clinical signs of underlying lung disease, we performed a chest X-ray with the anticipation that the patient might require biologics in the future. Chest X-ray (Figure 6) revealed prominent reticular shadows. Subclinical lung disease was suspected, and the patient was asked to perform a six-minute walk test that came out normal. Pulmonary function test revealed forced expiratory volume in 1 second to the forced vital capacity of the lungs (FEV1/FVC) 94.6% and 88.2% s/o restrictive pattern.

**Figure 7:**
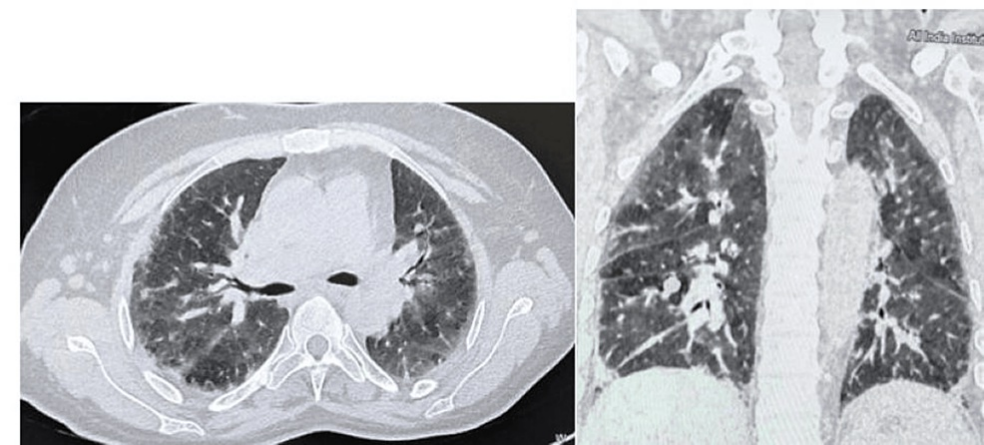
High-resolution CT scan of chest showing bilateral subpleural reticulations with ground glass opacities with sub-centimetric mediastinal lymph nodes with calcified lung nodules with surrounding cysts in bilateral upper lobes and right lower lobe suggestive of interstitial lung disease.

A high-resolution CT chest (Figure 7) showed bilateral subpleural reticulations with ground-glass opacities with subcentimetric mediastinal lymph nodes with calcified lung nodules with surrounding cysts in bilateral upper lobes and right lower lobe. This confirmed the presence of ILD that was clinically silent. Schirmer’s test revealed severe dry eyes (9 mm & 5 mm). Pilocarpine tablet was added for severe dry eyes. Nailfold capillaroscopy was done and revealed features of giant capillaries admixed with disorganized capillary architecture. Nifedipine tablet, 20 mg thrice a day, was started for Raynaud’s phenomenon.

**Figure 8:**
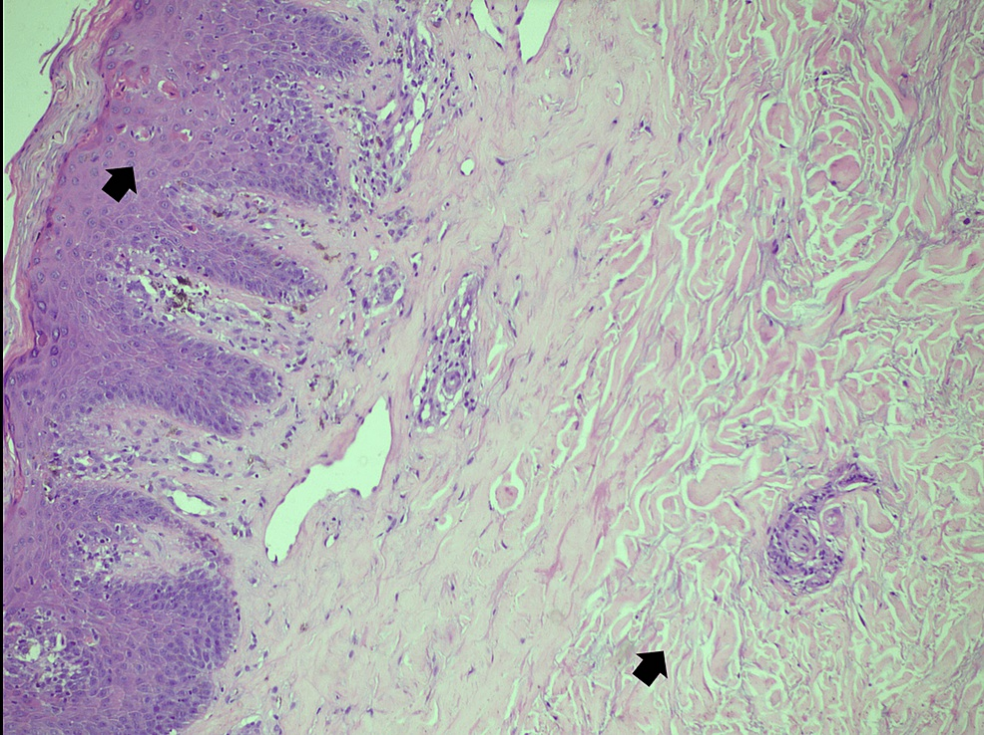
Medium power view of skin biopsy shows mild parakeratosis. Necrotic keratinocytes are seen (upper black arrow) with lymphocytic exocytosis. Papillary dermis shows mild chronic inflammation, dilated vessels, and pigment incontinence. Superficial dermis shows moderate perivascular inflammation and homogenization of collagen fibers. Lower arrow shows increased dermal mucin material. These features are consistent with cutaneous lupus.

**Figure 9:**
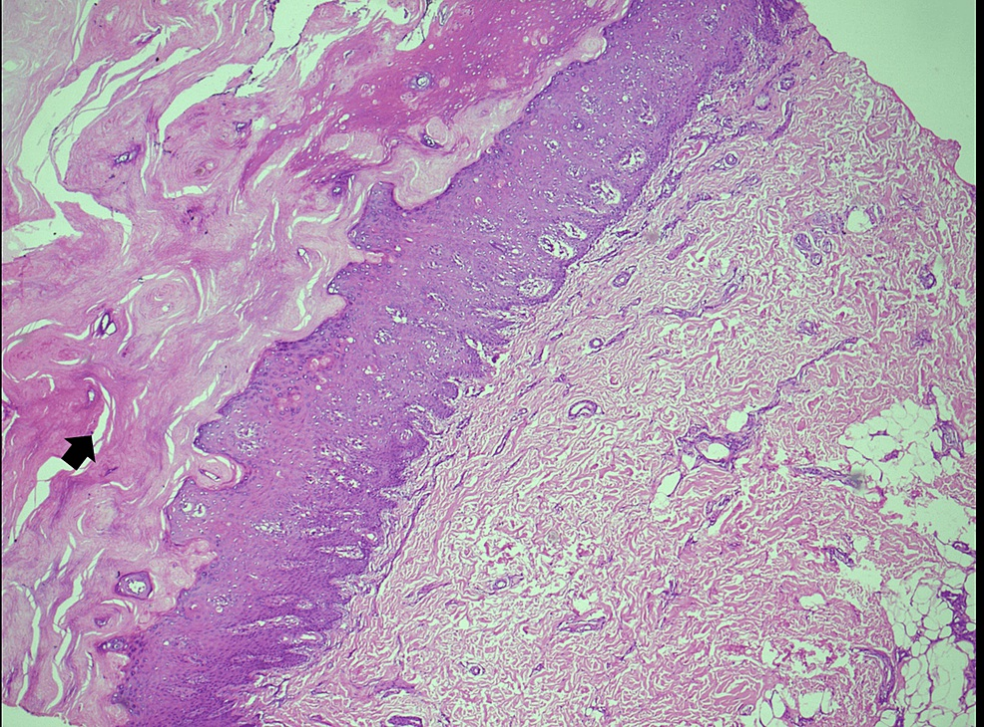
Low power view of skin biopsy. Black arrow shows extensive hyperkeratosis and orthokeratosis of the stratified squamous epithelium which is acanthotic and shows mild papillomatosis. Granular cell layer is seen prominently in focal areas. The rete ridges are elongated and interconnected. Mild inflammation is seen around superficial dermal plexus. Deeper dermis is showing few adnexal structures. These epidermal changes are indicative of keratoderma which occurs in dermatomyositis.

Skin biopsy from skin thickening over the arm was suggestive of cutaneous lupus erythematosus (Figure 8). This added information regarding overall diagnosis. Skin blackening and proximal muscle weakness started improving with the addition of steroids. The palmoplantar keratoderma was still refractory. After a few days, a second biopsy was taken from the sole that revealed the presence of keratoderma secondary to dermatomyositis (Figure 9).

**Figure 10:**
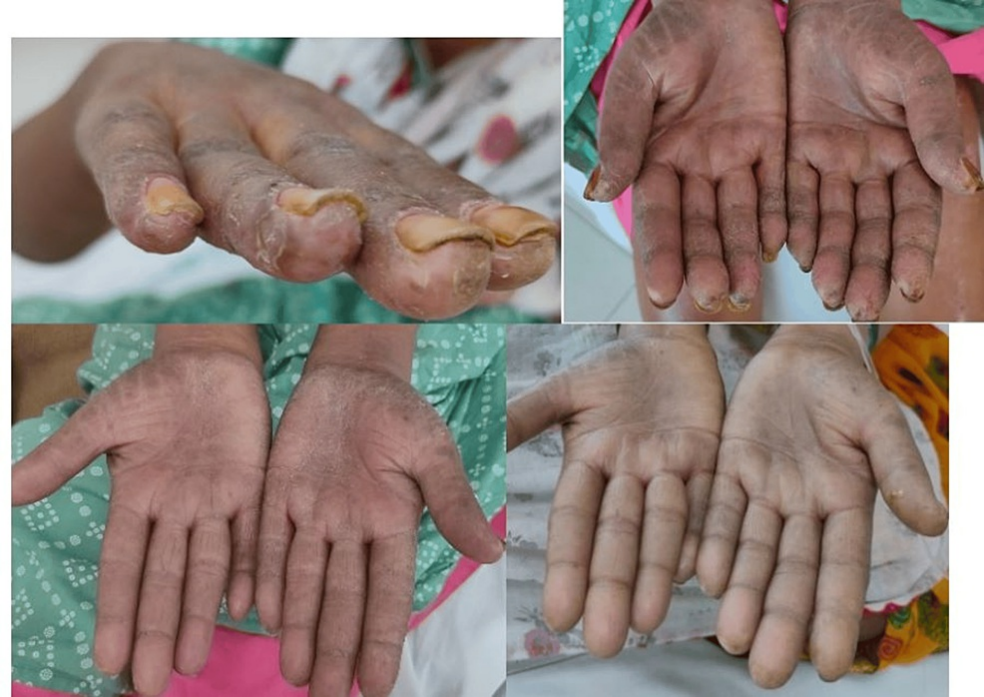
Sequential images showing improvement in skin plaques, nails, and fissures of both palms after starting treatment.

**Figure 11:**
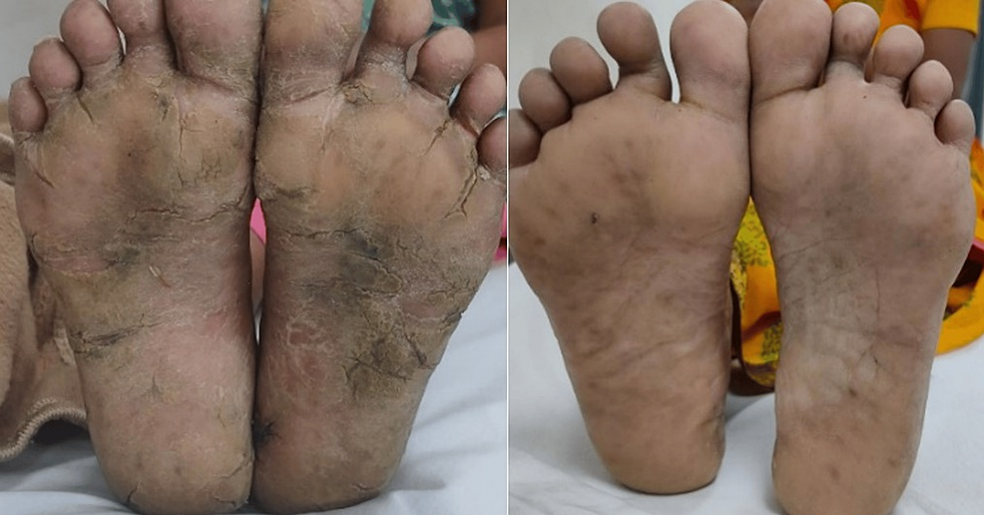
: Sequential images showing improvement in both soles after starting treatment.

Methotrexate tablet was started at a dose of 20 mg/week alongside topical tacrolimus. Dramatic improvement was seen in the keratoderma lesions in one month with clearance of scales and fissures (Figures 10, 11). Also, it was concluded that the erythematous lesions over the dorsum of the fingers were secondary to dermatomyositis and were not mechanic’s hands. Mechanic’s hands are typically found on the radial border of the fingers. Because there was no increase in total creatine phosphokinase (CPK), though there was proximal muscle weakness, a hypomyopathic variant of dermatomyositis was the most probable explanation. Final diagnosis was overlap syndrome with cutaneous lupus with secondary Sjögren’s with hypomyopathic dermatomyositis with palmoplantar keratoderma with U-ILD.

## Discussion

Anti-Golgi apparatus Ab is a rare Ab and has been associated with rheumatoid arthritis, Sjögren’s, and SLE [1]. It is a transient Ab and, if detected, must lead to suspicion of an underlying connective tissue disease. Dermatomyositis with normal CPK levels has been reported [2,3] and is associated with ILD, which is clinically aggressive and life-threatening. In our case, the ILD was probably in the early stages, and the patient was asymptomatic. Anti Jo-1, a marker of ILD, was negative. Although the nonspecific interstitial pneumonia (NSIP) pattern is most commonly reported in ILD associated with anti-PM/Scl-associated overlap syndrome, the pattern in our patient did not fit any particular known patterns and was thus classified as U-ILD. This shall be followed up radiologically.

Palmoplantar keratoderma is a rare presentation of dermatomyositis and was treated by Morrell et al. with methotrexate [4]. It has been reported in juvenile dermatomyositis by Patra et al. [5] and was successfully treated by standard treatment for dermatomyositis that included methotrexate. It was argued that palmoplantar keratoderma could be an exaggerated form of a mechanic’s hand. Antisynthetase syndrome could be a close differential diagnosis in our case, but the absence of antisynthetase antibodies and the presence of definitive histopathologic findings ruled out antisynthetase syndrome. The association of palmoplantar keratoderma has been described with the presence of anti-PM/Scl antibodies [5] and is also reported to be a rare entity in SLE, especially in patients positive for anti-Ro/SSA antibodies [6]. Anti-PM/Scl Ab are known to be associated with (in order of descending frequency) inflammatory myopathy/overlap syndrome/systemic sclerosis/undifferentiated CTD. ILD is the most common manifestation associated with the presence of anti-PM/Scl antibody. Other manifestations are Raynaud’s phenomenon, muscle weakness, and skin rashes [7].

## Conclusions

Our case report demonstrates significant heterogeneity in the ANA patterns, ILD patterns, and clinical manifestations as well as the treatment approaches. Palmoplantar keratoderma is a rarely reported finding in dermatomyositis and SLE. Inflammatory myopathy may have normal CPK levels. Inflammatory myopathy with normal CPK levels should be screened for ILD. Methotrexate is useful in treating keratoderma. An ANA with an anti-Golgi apparatus pattern may occur in SLE.
